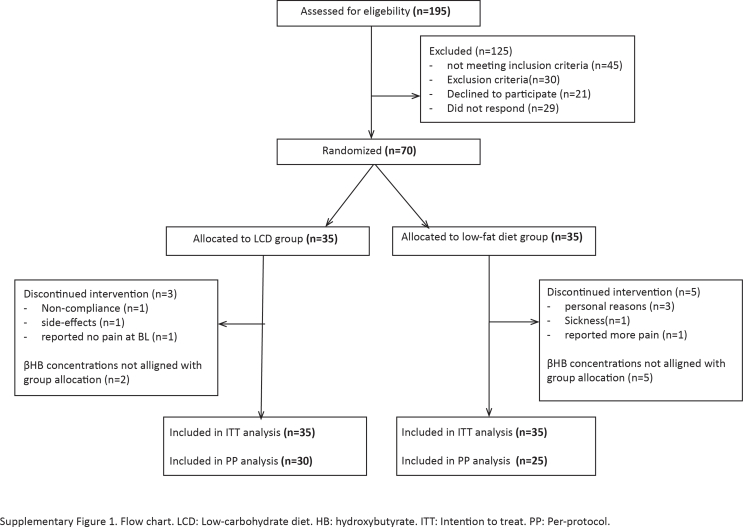# Changes in Cytokines and Fibrotic Growth Factors after Low-Carbohydrate or Low-Fat Low-Energy Diets in Females with Lipedema

**DOI:** 10.1016/j.cdnut.2025.104571

**Published:** 2025-02-20

**Authors:** Julianne Lundanes, Vilde Fiske Nes, Oda Aakervik, Liv Ryan, Patrik Hansson, Anne Mari Rokstad, Catia Martins, Siren Nymo

**Affiliations:** 1 Department of Clinical and Molecular Medicine, Faculty of Medicine and Health Sciences, Norwegian University of Science and Technology, Trondheim, Norway; 2 Nord-Trøndelag Hospital Trust, Clinic of Surgery, Namsos Hospital, Namsos, Norway; 3 Clinical Nutrition Research Group, Department of Clinical Medicine, UiT The Arctic University of Norway, Tromsø, Norway; 4 Centre for Nutrition, Department of Clinical Medicine, University of Bergen, Bergen, Norway; 5 Department of Food and Nutrition and Sport Science, University of Gothenburg, Gothenburg, Sweden; 6 Department of Nutrition Sciences, University of Alabama at Birmingham, Birmingham, AL, United States; 7 Department of Public Health and Nursing, Faculty of Medicine and Health Sciences, Norwegian University of Science and Technology, Trondheim, Norway; 8 Centre for Obesity and Innovation, Clinic of Surgery, St. Olav University Hospital, Trondheim, Norway

**Keywords:** inflammation, CRP, TNF, weight loss, ketogenic diet

## Abstract

**Background:**

Lipedema is considered an inflammation-related disease, and low-carbohydrate ketogenic diets may help reduce inflammation. However, no randomized controlled trials have investigated the effect of a low-carbohydrate ketogenic diet on inflammatory markers in females with lipedema.

**Objectives:**

To compare changes in inflammatory and fibrosis-associated markers after a low-energy low-carbohydrate diet (LCD) compared with a low-fat diet in females with lipedema, and to explore potential associations between changes in pain and changes in inflammatory and fibrosis-associated markers.

**Methods:**

Females with lipedema and obesity were randomly assigned to either an LCD or low-fat diet (both 1200 kcal/d) for 8 weeks. Body composition [fat mass (FM) and fat-free mass] and the plasma concentrations of high-sensitivity C-reactive protein (hsCRP), cytokines, and fibrosis-associated markers were measured pre- and postintervention.

**Results:**

A total of 70 females were included (35/group) (mean age: 47.3 ± 10.9 y, BMI: 36.9 ± 4.9 kg/m^2^). Both groups lost weight and FM (kg and %), with a greater reduction in the LCD group. A reduction in macrophage inflammatory protein-1ß, tumor necrosis factor-α, and hsCRP was seen in the LCD group only, despite no significant differences between groups. No associations were found between changes in pain and changes in cytokines and fibrosis-associated markers.

**Conclusions:**

Changes in cytokines and fibrosis-associated markers did not differ between low-energy LCD and low-fat diets in females with lipedema, despite a beneficial profile in the LCD group. Inflammation does not seem to be involved in pain reduction following LCD in this patient group.

**Trial registration number:**

This trial was registered at clinicaltrials.gov as NCT04632810.

## Introduction

Lipedema is characterized by painful fibrotic subcutaneous adipose tissue accumulation in the lower limbs in females [1,2]. It is a disease occurring in phases with reproductive hormonal fluctuations, such as puberty, pregnancy, and menopause, suggesting an estrogen-related etiology [[3], [4], [5]]. An altered estrogen receptor pattern may result in adipocyte hyperplasia and hypertrophy [4,5]. This can lead to hypoxia, which may, in turn, result in both neurogenic inflammation and tissue fibrosis and, in combination with the release of proinflammatory cytokines [4], microangiopathy [1,4]. However, the pathogenesis of lipedema is still not fully understood.

Localized inflammation in the lipedema-affected adipose tissue has been found in biopsies’ studies [1,[6], [7], [8], [9]]. However, systemic inflammation also seems to be present [[9], [10], [11]], with upregulated tumor necrosis factor (TNF)-α, vascular endothelial growth factor (VEGF)-A, and transforming growth factor (TGF)-β [11], as well as VEGF-C [9], IL-11, IL-28A, and IL-29 [10].

Ketogenic diets have been suggested to reduce inflammation [12]; however, the mechanisms remain unclear. Several hypotheses have been suggested, namely, weight and fat mass (FM) loss, energy restriction, and ketone bodies production [12]. A recent meta-analysis has shown that ketogenic diets may reduce the concentrations of TNF-α and IL-6 [12]. However, this has not been investigated in a population with both lipedema and obesity. Considering the inflammatory status in females with lipedema, this warrants further investigation, particularly in light of the potential for low-carbohydrate ketogenic duets to reduce pain in this patient group [13,14].

Therefore, the primary objective of this study was to compare changes in the plasma concentrations of high-sensitivity C-reactive protein (hsCRP), cytokines, and fibrotic growth factors after a low-energy low-carbohydrate diet (LCD) compared with a low-fat diet in females with lipedema, and to explore potential associations between changes in pain and changes in cytokines and fibrosis-associated markers. It was hypothesized that a larger reduction in hsCRP, cytokines, and fibrotic growth factors was seen in the LCD compared with the low-fat diet group, and that these changes were associated with changes in pain.

## Methods

### Study design

This is a secondary analysis of a randomized controlled trial (RCT) comparing a low-energy LCD with a low-fat low-energy (control) diet for 8 wk in female patients with lipedema [13]. The study was approved by the Regional Ethics Committee for Medical and Health Research Ethics of Central Norway (REK; 93888). All participants provided written informed consent in line with the Helsinki Declaration before entering the study. Participants were randomly assigned (1:1) by block randomization with stratification by BMI categories (30.0–34.9, 35.0–39.9, and 40.0–44.9 kg/m^2^). Randomization was performed by a web-based randomization system developed and administered by the Faculty of Medicine and Health Sciences, Norwegian University of Science and Technology. The data collection was done using eFORSK, a web-based system developed and administered by Helse Midt-Norge information technology (IT; Central Norway Regional Health Authority’s IT department).

### Study population

Inclusion criteria included a female sex, lipedema diagnosis by physiotherapist, age between 18 and 75 y, BMI between 30 and 45, and weight stability for the last 3 mo (± 3 kg). Participants were diagnosed with lipedema by physical therapists before inclusion, and type and stage of lipedema were assessed at baseline (BL). Exclusion criteria included acute and chronic kidney disease/failure, previous bariatric surgery, malignant disease, infectious disease, diabetes, psychosocial disorders, breastfeeding, pregnancy, current medication known to affect body weight, no mastery of a Scandinavian language, and enrollment in another obesity/lipedema treatment program (except for regular physical therapy). Those participants using pain medication and/or compression garments or pulsators should continue using them throughout the study.

### Dietary intervention

Diets were matched for energy (1200 kcal/d) and protein (60 g; energy percentage [E%], 20 E%) but differed in carbohydrate (CHO) and fat content. The LCD consisted of 75 g of CHO (25 E%) and 73 g of fat (55 E%), whereas the low-fat diet consisted of 180 g of CHO (60 E%) and 27 g of fat (20 E%). The dietary plans were adjusted with respect to food preferences, intolerances, and allergies. Participants were advised to take a multivitamin/mineral supplement and to drink a minimum of 2 L of energy-free drinks daily. For more information about the prescribed diets and energy and macronutrient intake in each diet group, see Lundanes et al. [13].

### Compliance

Participants had weekly follow-ups, either by phone or face-to-face, depending on convenience. Body weight was measured, ketosis was assessed (see “Ketosis” section), and potential side effects of the diets were discussed, aiming at enhancing compliance and preventing dropouts. Necessary changes and adjustments in the diets were made within the constraints of energy and macronutrient distribution.

Participants were asked to fill out daily food records throughout the intervention period. These were then analyzed for intake of energy (kcal per day) and macronutrients (grams per day, E%) using a web-based analysis program based on the Norwegian Food Composition Table and were discussed at the weekly follow-ups. Compliance data from this sample have already been published [13].

### Ketosis

Ketostix reagent test strips (Ascensia Diabetes Care Holdings AG) were used in the weekly follow-ups to measure urinary acetoacetate (AcAc) concentration. A cutoff concentration <0.5 mmol/L was used for negative AcAc. ß-hydroxybutyrate (ßHB) concentration was also measured in whole blood (FreeStyle Precision Neo, Abbott Laboratories) using finger pricks at BL, week 5, and week 9. These data have already been published [13]. Participants with a ßHB concentration ≥0.3 mmol/L were categorized as being in nutritional-induced ketosis [15]. In the per-protocol (PP) analysis, participants in the LCD group had to be ketotic at week 9, whereas participants in the low-fat group had to be nonketotic, based on ßHB concentrations to be included in the analyses. If borderline at week 9, ≥4 AcAc urine concentrations, in line with group allocation during the weekly follow-ups, were needed for inclusion in the PP analysis.

### Outcome measures

The following variables were assessed at BL and week 9 in the obesity outpatient clinic at St. Olavs University Hospital.

#### Body weight and body composition

Weight (kilograms) was measured with Seca 876 (SECA) to the nearest 0.1 kg. Body composition [FM and fat-free mass (FFM)] was assessed using dual-energy X-ray absorptiometry (Lunar, GE Healthcare).

#### Pain

Pain was assessed using brief pain inventory. Only the question assessing pain now (How much pain are you in right now?) was used in the present analysis. All pain variables from this sample have been previously published [13].

#### High-sensitivity C-reactive protein

A nonfasting blood sample was collected in a 4-mL EDTA tube and 11 μL of aprotinin was added. The tube was then centrifuged at 18 °C, for 10 min at 2106 G. The plasma was then retrieved and stored at −80 °C until analysis. HsCRP was analyzed by St. Olav Hospital HF, Laboratory Medicine Clinic, Department of Medical Biochemistry, using standardized methods.

#### Cytokines and fibrosis-associated markers

Fasting blood samples were collected in EDTA tubes and immediately (<5 min) centrifuged at 2500 G for 15 min at 4 °C. The EDTA plasma was then retrieved and stored at −80 °C until analysis. The cytokines and fibrosis-associated markers were analyzed at the Norwegian University of Science and Technology using a Bio-Plex Pro Human Cytokine 27-plex assay (Bio-Rad). The manufacturer’s protocol was followed with the recommended concentration of reagents and serum, but in reduced volume 1:2. The following cytokines were analyzed: Eotaxin, basic fibroblast growth factor (FGF-b), granulocyte colony stimulating factor, interferon (IFN)-γ, interferon γ-induced protein (IP)-10, IL-1b, IL-1 receptor antagonist (IL-1Ra), IL-2, IL-4, IL-5, IL-6, IL-7, IL-8, IL-9, IL-10, IL-12p70, IL-13, IL-15, IL-17, monocyte chemotactic protein-1, macrophage inflammatory protein (MIP)-1α, MIP-1ß, platelet-derived growth factor BB, RANTES, TNF-α, and VEGF-A. The following cytokines were not included due to high prevalence (>20 %) of out-of-range (OOR) values: FGF-b, IL-1ß, IL-1RA, IL-1, IL-5, IL-6, IL-12p70, IL-15, and VEGF. For variables with ≤20% OOR values, the OOR values were replaced by the lowest identified value on the plate divided by 2. Fibrosis-related cytokines were analyzed using Bio-Plex Pro TGF-β 3-plex assay (Bio-Rad). The following markers were analyzed: TGF-ß1, TGF-ß2, and TGF-ß3. Both time points from the same participants were analyzed on the same plate, and the 2 diet groups were equally distributed on each plate.

#### Statistical analysis

Statistical analysis was performed using Stata (StataCorp LLC version 18), and data were presented as means ± SD or estimated marginal means with a corresponding 95% confidence interval. Residuals were checked for normality with a Shapiro–Wilk test and visual inspection of histograms and Q–Q plots. Statistical significance was assumed at *P* < 0.01 due to the large number of outcome variables, to account for an increased risk of type 1 error. Group differences in the changes from BL were estimated by linear mixed-effect models. The fixed part was specified in terms of 2 dummy variables: one for time and one for group differences (LCD compared with low-fat diet) post intervention (week 9) because the BL means can be assumed to be the same given the randomly assigned design [16,17]. The mean difference in changes from BL is equivalent to the estimated mean group difference postintervention. A random intercept for patient was included to account for within-patient correlations. Pearson or spearman correlation, depending on the normality of data, was used to investigate the associations between changes in pain and changes in hsCRP, cytokines, and fibrosis-associated markers. Figures were generated using GraphPad Prism (version 10.0.2 for Windows, GraphPad Software).

Intention-to-treat (ITT) analysis was performed with all included participants, whereas PP analysis included only participants who were compliant with the interventions (as previously described).

## Results

### Study population

A total of 70 female participants with lipedema and obesity were included in this study, 35 in each group (see flow chart in Supplemental Figure 1), with an average age of 47.3 ± 10.9 y and a BMI of 36.9 ± 4.9 kg/m^2^. Participants’ characteristics at BL are presented in Table 1.

**TABLE 1:** Baseline characteristics of the participants.

	All participants (*n* = 70)	LCD (*n* = 35)	Control diet (*n* = 35)
	Mean ± SD	Mean ± SD	Mean ± SD
Age, (y)	47.3 ± 10.9	48.4 ± 8.9	46.2 ± 12.6
BMI, (kg/m^2^)	36.9 ± 4.9	36.7 ± 4.6	37.1 ± 5.3
Weight, (kg)	103.2 ± 14.6	101.2 ± 13.7	104.2 ± 15.6
Height, (cm)	167.2 ± 6.1	167.0 ± 6.6	167.4 ± 5.8

Data presented as mean ± SD.

Abbreviations: LCD, low-carbohydrate diet.

### Body weight and body composition

Body weight and body composition over time are presented in Table 2. Both groups experienced a significant reduction in body weight, FM (both kg and %) and FFM (all *P* < 0.001); however, the reduction in FM (kg and %) was greater in the LCD group compared with the low-fat diet group (*P* = 0.005 and *P* = 0.001, respectively).

**TABLE 2:** Body weight and body composition before and after low-carbohydrate and low-fat diets.

	BL	Week 9	Difference from BL to week 9			Difference in change between groups		
	Mean ± SD	Mean ± SD	EMM	95% CI	*P* value	EMM	95% CI	*P* value
Weight, (kg)								
LCD	102.3 ± 13.6	92.1 ± 13.4	−10.2	−11.2 to −9.4	<0.001	−2.9	−4.0 to −1.7	<0.001
Low-fat diet	104.1 ± 15.6	94.9 ± 15.8	−7.3	−8.2 to −6.5	<0.001			
FM Total, (kg)								
LCD	50.7 ± 8.9	42.6 ± 8.0	−7.0	−8.0 to −6.1	<0.001	−2.0	−3.3 to −0.6	0.005
Low-fat diet	52.9 ± 10.1	45.4 ± 9.0	−5.1	−6.1 to −4.1	<0.001			
FFM Total, (kg)								
LCD	51.1 ± 5.5	48.1 ± 4.9	−2.5	−3.0 to −2.0	<0.001	−0.2	−0.9 to 0.58	0.642
Low-fat diet	51.9 ± 5.4	48.7 ± 5.0	−2.3	−2.9 to −1.8	<0.001			
FM Total, (%)								
LCD	49.6 ± 3.3	46.7 ± 3.9	−2.7	−3.1 to −2.2	<0.001	−1.1	−1.8 to −0.4	0.001
Low-fat diet	49.7 ± 3.1	48.0 ± 3.4	−1.5	−2.0 to −1.1	<0.001			

Data presented as mean± SD, and changes within and between groups are presented as estimated marginal means and 95% CI.

Abbreviations: EMM, estimated marginal means; FM, fat mass; FFM, fat-free mass; LCD, low-carbohydrate diet.

### High-sensitivity C-reactive protein, cytokines, and fibrosis-associated markers

**FIGURE 1:**
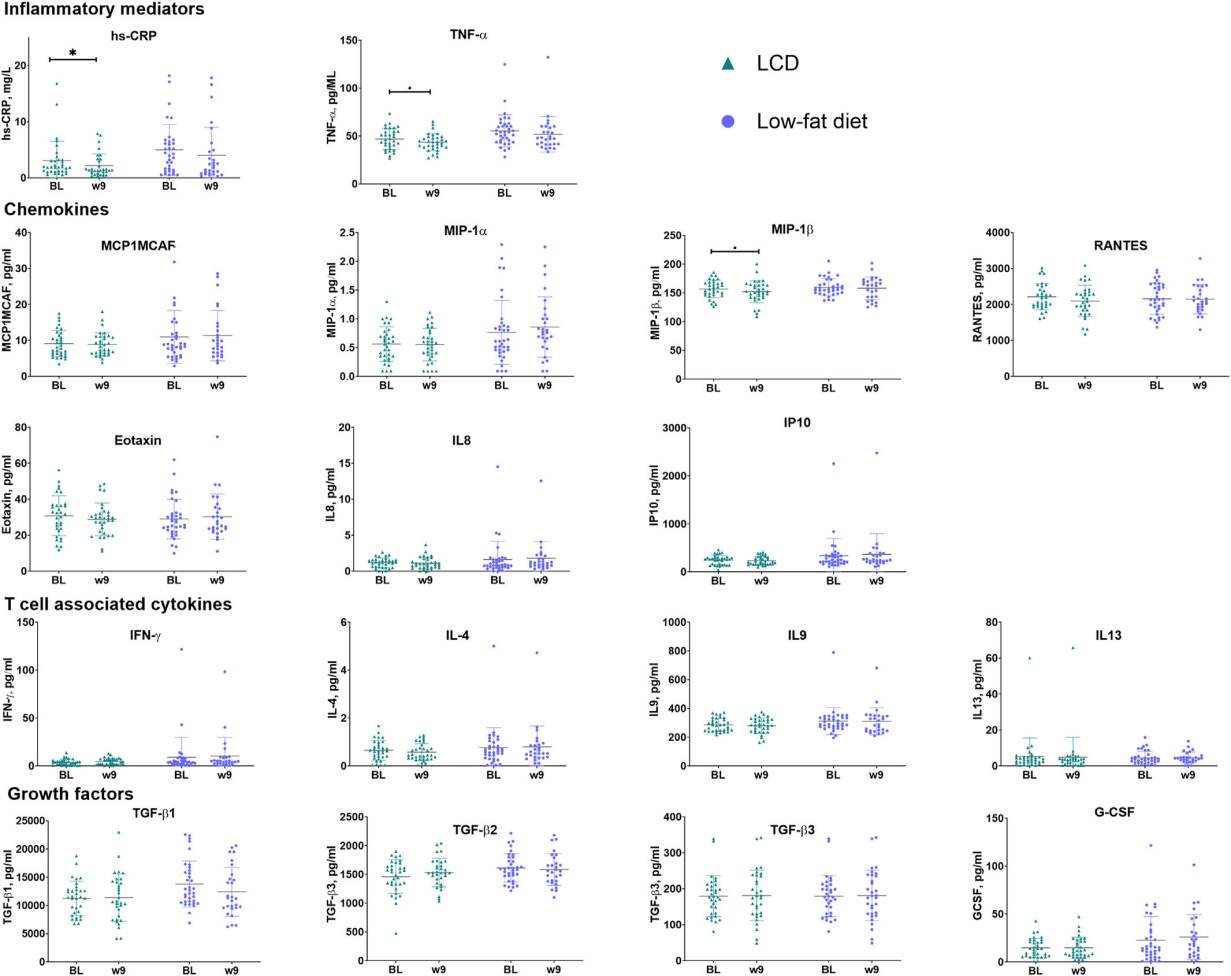
Plasma concentrations of hsCRP, cytokines, and fibrosis-related growth factors before and after low-carbohydrate and low-fat diets. Data presented as individual values, mean, and SD. ∗*P* < 0.01, Significant change from baseline to week 9 within group. *Abbreviations:* G-CSF, granulocyte colony stimulating factor; hsCRP, high-sensitivity C-reactive protein; IFN, interferon; LCD, low-carbohydrate diet; MIP, monocyte chemotactic protein; TGF, transforming growth factor; TNF, tumor necrosis factor.

HsCRP, cytokines, and fibrosis-associated cytokines over time can be seen in Figure 1 and Supplemental Table 1. There was a reduction in hsCRP (*P* = 0.004), the chemotactic cytokine MIP-1ß (*P* = 0.009), as well as the proinflammatory cytokine TNF-α (*P* = 0.005) in the LCD group, but no differences between groups were found.

No associations between changes in cytokines and fibrosis-associated markers and changes in pain and ketosis at week 9 were seen (see Supplemental Tables 2 and 3, respectively).

#### Per-protocol analysis

HsCRP, cytokines, and fibrosis-related growth factors for PP analysis are presented in Supplemental Table 4. These analyses revealed similar results as the ITT analysis. No associations between changes in hsCRP, cytokines, and fibrosis-associated markers and changes in pain and ketosis at week 9 were found (data not shown).

## Discussion

The primary objective of this study was to compare changes in plasma concentrations of hsCRP, cytokines, and fibrotic growth factors after a low-energy LCD compared with a low-fat diet in females with lipedema, and to determine potential associations between changes in pain and changes in cytokines, fibrosis-associated markers, and ketosis. A reduction in hsCRP, MIP-1ß, and TNF-α was seen in the LCD group; however, no significant differences between groups were found. Moreover, no associations were found between changes in hsCRP, cytokines, and fibrosis-associated markers and changes in pain and ketosis at week 9.

A reduction in hsCRP was found in the LCD group only. HsCRP is produced in the liver as a response to increased levels of IL-6 and IL-1 and is a sensitive marker for inflammation-associated conditions [18]. A higher stage of lipedema is associated with higher concentrations of hsCRP, possibly attributed to higher prevalence of obesity [19]. In a case study by Cannataro et al. (2021), a reduction in CRP from 0.6 to 0.2 mg/dL was seen after a ketogenic diet for 7 mo, and to 0.1 after 21 mo. This was also found in our study, with a reduction of 1.4 mg/L (45 %) in the LCD group. A meta-analysis found that LCDs were significantly associated with decreases in plasma concentrations of CRP [20]. This suggests that the lack of differences between diet groups in this study might be due to power constraints.

The lack of between-group differences may also be due to the control group used, as well as the duration of trial. A reduction in hsCRP levels was also seen in the low-fat diet group, despite not significantly (−0.9 mg/L, *P* = 0.086). This suggests that WL may play a role in the observed reduction in hsCRP, and that a control group without weight loss (WL) would most likely translate into significant between-group differences in hsCRP. A meta-analysis comparing LCDs with low-fat diets found a beneficial effects of LCD on CRP [21], but this effect disappeared when the analysis was adjusted for energy intake. However, no direct association was found between CRP and WL, suggesting that WL is not the only mechanism involved [21]. The reduction in CRP was especially evident in trials with long duration [21], suggesting that the duration of this study may have been insufficient to detect significant differences between groups.

A reduction of the proinflammatory cytokine TNF-α was also found in the LCD group only. TNF-α is associated with oxidative stress [22], a condition present in lipedema [11,23]. Moreover, TNF-α concentrations have been found to be increased in patients with lipedema compared with BMI-matched controls without lipedema [11]. The same study also found higher concentrations of other oxidative stress markers in patients with lipedema, such as superoxide dismutase activity, catalase activity, and malondialdehyde concentrations, compared with a control group without lipedema [11]. The plasma concentrations of TNF-α in this study were substantially higher at BL (47 ± 11 and 56 ± 17 pg/mL in LCD and low-fat diet group, respectively) compared with the concentrations reported by Nankam et al., where females with lipedema exhibited concentrations of 2 ± 1 pg/mL. The participants had a lower BMI (32.5 ± 5.8) [11] compared with this study, which may account for discrepancies in TNF-α concentrations. However, it is also likely that assay differences and variations in sensitivity could explain these differences. It is known that TNF-α is elevated in individuals with obesity [[24], [25], [26], [27]], and weight loss has been shown to reduce TNF-α concentrations [28]. Thus, we cannot exclude the possibility of a “double burden” of both lipedema and obesity; however, this needs to be further investigated.

The lack of between-group differences may result in the fact that the low-fat diet group also had a significant weight loss. Moreover, a meta-analysis of RCTs found no effect of LCD compared with a low-fat diet on TNF-α, neither for individuals with obesity nor in short- or long-term interventions [21]. It may be speculated that the effect on TNF-α may be specific for lipedema, as this marker has been shown to be elevated in lipedema compared with BMI-matched controls [11].

Recent studies have shown that ketogenic diets might have anti-inflammatory properties and may modulate the concentrations of inflammatory markers [[29], [30], [31]], whereas other studies have found no effect [29,32,33]. Pooled estimates from a meta-analysis by Ji et al. revealed a significant reduction in TNF-α and IL-6 after a ketogenic diet [12]. Moreover, the reduction in TNF-α was more pronounced in short-term studies (≤8 wk), and IL-6 concentrations decreased to a greater extent after following a ketogenic diet in participants with obesity compared with individuals with BMI < 30 [12]. Unfortunately, IL-6 concentrations were below the detection limit in this study; hence, we were unable to measure this cytokine. The reduction in TNF-α was in line with the meta-analysis from Ji et al. [12].

The mechanisms through which ketogenic diets might reduce inflammation remain inconsistent; however, ketosis is one potential alternative [12]. ßHB can bind to specific receptors in the adipocytes and immune cells, known as hydroxycarboxylic acid receptors. When ßHB activates those receptors, a reduction in the release of TNF-α and IL-6 occurs [12]. Verde et al. [34] argued that a very low-energy ketogenic diet can reduce inflammation in patients with lipedema through nutritional-induced ketosis [34]. Despite this, no associations were found between TNF-α and ßHB in this present analysis. This lack of association between TNF-α and ßHB could be attributed to possibly insufficient statistical power, or it might indicate that the reduction in TNF-α is influenced by other factors, such as CHO intake, which has been shown to positively correlate with TNF-α concentrations [35].

Another potential mechanism for the reduction in inflammation seen with ketogenic diets is weight loss [12]. Weight loss leads to reduction in FM, with concomitant reductions in TNF-α and IL-6, which are produced in the adipose tissue [36]. Leptin may also increase inflammation by stimulating immune cells to produce TNF-α [37]. Obesity is associated with a chronic low-grade inflammation, and inflammatory markers related to obesity include TNF-α [[24], [25], [26], [27]], IL-6 [24,26,27,[38], [39], [40]], and the anti-inflammatory cytokine IL-10 [27]. An average weight loss of 19% induced by a very low-energy diet for 12 or 16 wk has been shown to reduce TNF-α, IL-1b, IL-6, IL-12, IL-23, and IFN-γ [41]. It has been argued that a weight loss of >10% is needed for improvements in inflammatory markers to be seen in individuals with obesity [42]. A WL of 10% was seen in the LCD group and may explain why a reduction in hsCRP, TNF-α, and MIP-1β was seen in this group only. Energy restriction per se has also been shown to have an anti-inflammatory effect in individuals with obesity; however, all studies have investigated individuals with obesity and elevated inflammatory markers. Hence, it needs to be investigated whether the effects are independent of weight loss or not [12]. The anti-inflammatory effects of ketogenic diets might also be driven by adenosine levels and gut microbiota [12]; however, these were not investigated in this study.

Contrary to our hypotheses, few differences were observed both within and between groups. The low overall changes in cytokines seen in the LCD group support the theory of a more localized inflammation and less systemic pathological inflammation. Another issue could be the selection of cytokines and fibrosis-associated markers in this study. Several of the cytokines found to be elevated in females with lipedema, for example, VEGF-A [11], VEGF-C [9], IL-11, IL-28A, and IL-29 [10], were below the detection limit or not included in the analysis kit in this study. Thus, we did not succeed in determining changes in these cytokines.

No associations were found between changes in pain and changes in hsCRP, cytokines, and fibrosis-associated markers. Even though one of the hypotheses regarding pain reduction following LCD in this patient group is reduced inflammation, the pain pathogenesis in patients with lipedema remains inconclusive [3] and is most likely multifactorial. Furthermore, prostaglandins, bradykinin, and leukotrienes should be further investigated in relation to lipedema, as they have been shown to mediate pain signals [[43], [44], [45]].

This study has both strengths and weaknesses. From our knowledge, this is the first RCT investigating the effect of an LCD on hsCRP, cytokines, and fibrosis-associated markers in females with lipedema. A broad spectrum of cytokines was analyzed, and there was a low drop-out rate and strong dietary compliance. However, there are also some limitations. The present analyses are exploratory by nature, as the study was powered to investigate pain [13], not inflammation. Hence, the statistical power may be too low to detect differences in cytokines between groups. This also raises the issue of multiple testing, and even though a stricter *P* value was used, the results need to be interpreted with caution. Moreover, the cytokines were analyzed using a 27-plex, which lowers the analytic precision, and several cytokines were below the detection limit. However, this field of research remains largely unexplored; thus, the present findings provide valuable information that may guide further research.

In conclusion, changes in cytokines and fibrosis-associated markers did not differ between low-energy LCD and low-fat diets in females with lipedema, despite a beneficial profile in the LCD group. Changes in systemic Inflammation do not seem to be involved in pain reduction following LCD in this patient group. Further studies should target localized inflammation in the lipedema-affected adipose tissue.

## Author contributions

The authors’ responsibilities were as follows – SN, CM, AMR, JL: designed the study and formulated the research question; JL, VFN, OA: carried out the study; LR: analyzed the cytokines and fibrosis-associated markers; JL: analyzed the data; AMR, PH, CM, SN, JL: were involved with interpretation of the results; and all authors: were involved in writing up the manuscript.

## Data availability

Data described in the manuscript will be available upon request, pending approval from the Ethics Committee.

## Funding

This study was supported by the Norwegian women’s public health association (Women’s health research program), the Nord-Trøndelag Hospital Trust, and the Norwegian Lymphoedema and Lipedema Association. The funding sources had no involvement or restrictions regarding publication.

## Conflict of interest

The authors declare no conflict of interest.

## Figures and Tables

**Figure:**