# Supplementary material for: Changes in Cytokines and Fibrotic Growth Factors after Low-Carbohydrate or Low-Fat Low-Energy Diets in Females with Lipedema
Source: Curr Dev Nutr. 2025 Feb 20;9(3):104571. doi: 10.1016/j.cdnut.2025.104571 (PMC11929874; doi:10.1016/j.cdnut.2025.104571)

# Manuscript: Changes in cytokines and fibrotic growth factors after low-carbohydrate or low-fat low-energy diets in females with lipedema

First Author: Julianne Lundanes

| Supplementary table 1. Systemic cytokine concentrations and fibrosis markers before and after low-carbohydrate or low-fat diets and changes within and between groups. | | | | | | | | |
| --- | --- | --- | --- | --- | --- | --- | --- | --- |
|  | **BL** | **W9** | **Difference from BL to w9** | | | **Difference in change between groups** | | |
|  | **Mean ± SD** | **Mean ± SD** | **EMM** | **95% CI** | **P value** | **EMM** | **95% CI** | **P value** |
| hsCRP, mg/L |  |  |  |  |  |  |  |  |
| LCD | 3.1 ± 3.5 | 2.2 ± 2.1 | -1.4 | -2.3 to -0.4 | **0.004** | -0.5 | -1.8 to 0.8 | 0.441 |
| Low-fat diet | 5.0 ± 4.5 | 4.0 ± 5.0 | -0.9 | -1.8 to 0.1 | 0.086 |  |  |  |
| TGF-ß1, pg/ml |  |  |  |  |  |  |  |  |
| LCD | 11256.0 ± 3016.1 | 11388.4 ± 4195.6 | -872.6 | -2366.9 to 621.7 | 0.252 | -320.9 | -2266.9 to 1625.1 | 0.747 |
| Low-fat diet | 13799.3 ± 4081.0 | 12447.5 ± 4333.4 | -551.7 | -2117.6 to 1014.2 | 0.490 |  |  |  |
| TGF-ß2, pg/ml |  |  |  |  |  |  |  |  |
| LCD | 1459.7 ± 294.4 | 1531.9 ± 248.4 | 17.8 | -80.5 to 116.0 | 0.723 | 6.4 | -122.8 to 135.5 | 0.923 |
| Low-fat diet | 1613.2 ± 246.5 | 1584.2 ± 277.6 | 11.4 | -91.5 to 114.3 | 0.828 |  |  |  |
| TGF-ß3, pg/ml |  |  |  |  |  |  |  |  |
| LCD | 179.1 ± 56.9 | 181.1 ± 70.3 | -15.7 | -40.8 to 9.4 | 0.221 | -11.6 | -44.7 to 21.5 | 0.492 |
| Low-fat diet | 224.9 ± 66.0 | 207.2 ± 78.8 | -4.1 | -30.4 to 22.2 | 0.760 |  |  |  |
| Eotaxin, pg/ml |  |  |  |  |  |  |  |  |
| LCD | 30.8 ± 11.1 | 29.0 ± 9.1 | -2.2 | -4.6 to 0.1 | 0.060 | -2.9 | -6.2 to 0.4 | 0.093 |
| Low-fat diet | 28.9 ± 11.0 | 30.3 ± 12.6 | 0.6 | -1.9 to 3.1 | 0.627 |  |  |  |
| G-CSF, pg/ml |  |  |  |  |  |  |  |  |
| LCD | 14.7 ± 10.0 | 14.7 ± 10.9 | -0.2 | -3.3 to 3.0 | 0.914 | -3.5 | -8.0 to 1.0 | 0.129 |
| Low-fat diet | 22.5 ± 24.7 | 26.1 ± 23.5 | 3.3 | -0.0 to 6.7 | 0.052 |  |  |  |
| IFN-γ, pg/ml |  |  |  |  |  |  |  |  |
| LCD | 3.7 ± 3.2 | 4.5 ± 3.4 | 0.7 | -1.0 to 2.4 | 0.420 | 0.3 | -2.2 to 2.9 | 0.784 |
| Low-fat diet | 8.9 ± 20.9 | 10.3 ± 19.5 | 0.4 | -1.5 to 2.2 | 0.704 |  |  |  |
| IL-4, pg/ml |  |  |  |  |  |  |  |  |
| LCD | 0.6 ± 0.4 | 0.6 ± 0.4 | -0.1 | -0.2 to 0.1 | 0.354 | -0.0 | -0.2 to 0.1 | 0.636 |
| Low-fat diet | 0.8 ± 0.8 | 0.9 ± 0.9 | -0.0 | -0.2 to 0.1 | 0.817 |  |  |  |
| IL-8, pg/ml |  |  |  |  |  |  |  |  |
| LCD | 1.2 ± 0.7 | 1.1 ± 0.8 | -0.1 | -0.3 to 0.2 | 0.641 | -0.2 | -0.5 to 0.2 | 0.399 |
| Low-fat diet | 1.6 ± 2.5 | 1.8 ± 2.3 | 0.1 | -0.2 to 0.4 | 0.480 |  |  |  |
| IL-9, pg/ml |  |  |  |  |  |  |  |  |
| LCD | 286.1 ± 45.7 | 280.2 ± 53.3 | -14.4 | -30.1 to 1.2 | 0.070 | -14.6 | -37.0 to 8.0 | 0.201 |
| Low-fat diet | 310.5 ± 93.8 | 309.9 ± 94.8 | 0.2 | -16.6 to 17.0 | 0.983 |  |  |  |
| IL-13, pg/ml |  |  |  |  |  |  |  |  |
| LCD | 5.3 ± 10.2 | 4.8 ± 11.1 | -0.4 | -1.2 to 0.4 | 0.369 | -0.3 | -1.5 to 0.9 | 0.608 |
| Low-fat diet | 4.4 ± 3.7 | 4.6 ± 3.2 | -0.1 | -0.9 to 0.8 | 0.888 |  |  |  |
| IP-10, pg/ml |  |  |  |  |  |  |  |  |
| LCD | 245.3 ± 99.0 | 230.6 ± 87.8 | -17.1 | -43.9 to 9.7 | 0.210 | -25.6 | -64.7 to 13.5 | 0.200 |
| Low-fat diet | 333.1 ± 363.5 | 362.7 ± 438.0 | 8.5 | -20.3 to 37.2 | 0.564 |  |  |  |
| MCP-1, pg/ml | |  |  |  |  |  |  |  |
| LCD | 9.1 ± 3.7 | 8.8 ± 3.3 | -0.8 | -1.9 to 0.4 | 0.194 | -1.2 | -2.9 to 0.4 | 0.134 |
| Low-fat diet | 10.9 ± 7.4 | 11.3 ± 7.0 | 0.5 | -0.7 to 1.7 | 0.428 |  |  |  |
| MIP-1α, pg/ml |  |  |  |  |  |  |  |  |
| LCD | 0.5 ± 0.3 | 0.5 ± 0.3 | -0.0 | -0.1 to 0.1 | 0.683 | -0.1 | -0.2 to 0.0 | 0.063 |
| Low-fat diet | 0.8 ± 0.6 | 0.9 ± 0.5 | 0.2 | 0.0 to 0.2 | 0.035 |  |  |  |
| MIP-1ß, pg/ml |  |  |  |  |  |  |  |  |
| LCD | 156.8 ± 15.3 | 151.9 ± 19.2 | -6.7 | -11.7 to -1.7 | **0.009** | -5.8 | -12.8 to 1.2 | 0.103 |
| Low-fat diet | 159.7 ± 14.8 | 158.0 ± 19.7 | -0.9 | -6.3 to 4.5 | 0.750 |  |  |  |
| RANTES, pg/ml |  |  |  |  |  |  |  |  |
| LCD | 2212.7 ± 366.2 | 2094.3 ± 438.1 | -92.4 | -211.9 to 27.1 | 0.129 | -106.4 | -273.1 to 60.4 | 0.211 |
| Low-fat diet | 2154.6 ± 434.9 | 2146.3 ± 408.7 | 13.9 | -114.8 to 142.6 | 0.832 |  |  |  |
| TNF-α, pg/ml |  |  |  |  |  |  |  |  |
| LCD | 46.8 ± 11.0 | 43.5 ± 8.9 | -5.0 | -8.5 to -1.5 | **0.005** | -3.1 | -8.1 to 1.9 | 0.220 |
| Low-fat diet | 55.8 ± 16.8 | 52.7 ± 18.6 | -1.9 | -5.6 to 1.9 | 0.325 |  |  |  |
| Data presented as mean ± SD, and results from linear mixed models are presented as estimated marginal means (EMM), 95% CI and p value. LCD: low-fat diet. hsCRP: High-sensitivity c-reactive protein. TGF: Transforming growth factor. G-CSF: Granulocyte colocy stimulating factor. IFN: interferon. IL: interleukin. MIP: monocyte chemotactic protein. TNF: Tumor necrosis factor. | | | | | | | | |

| Supplementary Table 2. Associations between changes in hsCRP, cytokines and fibrosis associated markers and Pain now at week 9. | | | |
| --- | --- | --- | --- |
|  | **All participants (n=70)** | **Low-fat diet (n=35)** | **LCD (n=35)** |
| △hsCRP | △**Pain now** | △**Pain now** | △**Pain now** |
| r | -0.091 | -0.321 | 0.108 |
| P value | 0.493 | 0.097 | 0.564 |
| △TGF-ß1 |  |  |  |
| r | -0.229 | -0.201 | -0.200 |
| P value | 0.084 | 0.304 | 0.288 |
| △TGF-ß2 |  |  |  |
| r | 0.032 | 0.098 | 0.098 |
| P value | 0.814 | 0.619 | 0.608 |
| △TGF-ß3 |  |  |  |
| r | -0.139 | -0.101 | -0.152 |
| P value | 0.297 | 0.609 | 0.427 |
| △Eotaxin |  |  |  |
| r | -0.161 | -0.336 | -0.210 |
| P value | 0.232 | 0.086 | 0.265 |
| △G-CSF |  |  |  |
| r | 0.163 | 0.357 | -0.049 |
| P value | 0.226 | 0.068 | 0.797 |
| △IFN-γ |  |  |  |
| r | -0.090 | 0.006 | -0.164 |
| P value | 0.505 | 0.978 | 0.386 |
| △IL-4 |  |  |  |
| r | -0.122 | -0.045 | -0.193 |
| P value | 0.366 | 0.824 | 0.307 |
| △IL-8 |  |  |  |
| r | -0.010 | -0.186 | 0.071 |
| P value | 0.940 | 0.354 | 0.710 |
| △IL-9 |  |  |  |
| r | -0.197 | -0.169 | -0.354 |
| P value | 0.142 | 0.401 | 0.055 |
| △IL-13 |  |  |  |
| r | 0.012 | -0.085 | 0.012 |
| P value | 0.930 | 0.674 | 0.951 |
| △IP-10 |  |  |  |
| r | -0.064 | -0.132 | -0.099 |
| P value | 0.638 | 0.513 | 0.601 |
| △MCP-1 |  |  |  |
| r | 0.010 | 0.110 | -0.185 |
| P value | 0.943 | 0.587 | 0.327 |
| △MIP-1α |  |  |  |
| r | 0.135 | 0.162 | 0.006 |
| P value | 0.317 | 0.420 | 0.977 |
| △MIP-1β |  |  |  |
| r | -0.1401 | -0.079 | -0.361 |
| P value | 0.297 | 0.696 | 0.050 |
| △RANTES |  |  |  |
| r | 0.020 | -0.036 | -0.045 |
| P value | 0.883 | 0.860 | 0.815 |
| △TNF-α |  |  |  |
| r | -0.156 | -0.207 | -0.177 |
| P value | 0.246 | 0.230 | 0.349 |
| Data presented as Pearson correlation coefficient (r) and p value. hsCRP: High-sensitivity c-reactive protein. TGF: Transforming growth factor. G-CSF: Granulocyte colocy stimulating factor. IFN: interferon. IL: interleukin. MIP: monocyte chemotactic protein. TNF: Tumor necrosis factor. | | | |

| Supplementary Table 3. Associations between changes in hsCRP, cytokines and fibrosis associated markers and βHB at week 9. | | | |
| --- | --- | --- | --- |
|  | **All participants (n=70)** | **Low-fat diet (n=35)** | **LCD (n=35)** |
| △hsCRP | **△ βHB** | **△ βHB** | **△ βHB** |
| r | -0.091 | -0.321 | 0.108 |
| P value | 0.493 | 0.097 | 0.564 |
| △TGFß1 |  |  |  |
| r | -0.229 | -0.201 | -0.200 |
| P value | 0.084 | 0.304 | 0.288 |
| △TGFß2 |  |  |  |
| r | 0.032 | 0.098 | 0.098 |
| P value | 0.814 | 0.619 | 0.608 |
| △TGFß3 |  |  |  |
| r | -0.139 | -0.101 | -0.152 |
| P value | 0.297 | 0.609 | 0.427 |
| △Eotaxin |  |  |  |
| r | -0.161 | -0.336 | -0.210 |
| P value | 0.232 | 0.086 | 0.265 |
| △G-CSF |  |  |  |
| r | 0.163 | 0.357 | -0.049 |
| P value | 0.226 | 0.068 | 0.797 |
| △IFNγ |  |  |  |
| r | -0.090 | 0.006 | -0.164 |
| P value | 0.505 | 0.978 | 0.386 |
| △IL4 |  |  |  |
| r | -0.122 | -0.045 | -0.193 |
| P value | 0.366 | 0.824 | 0.307 |
| △IL8 |  |  |  |
| r | -0.010 | -0.186 | 0.071 |
| P value | 0.940 | 0.354 | 0.710 |
| △IL9 |  |  |  |
| r | -0.197 | -0.169 | -0.354 |
| P value | 0.142 | 0.401 | 0.055 |
| △IL13 |  |  |  |
| r | 0.012 | -0.085 | 0.012 |
| P value | 0.930 | 0.674 | 0.951 |
| △IP10 |  |  |  |
| r | -0.064 | -0.132 | -0.099 |
| P value | 0.638 | 0.513 | 0.601 |
| △MCP1 |  |  |  |
| r | 0.010 | 0.110 | -0.185 |
| P value | 0.943 | 0.587 | 0.327 |
| △MIP-1α |  |  |  |
| r | 0.135 | 0.162 | 0.006 |
| P value | 0.317 | 0.420 | 0.977 |
| △MIP-1β |  |  |  |
| r | -0.1401 | -0.079 | -0.361 |
| P value | 0.297 | 0.696 | 0.050 |
| △RANTES |  |  |  |
| r | 0.020 | -0.036 | -0.045 |
| P value | 0.883 | 0.860 | 0.815 |
| △TNFα |  |  |  |
| r | -0.156 | -0.207 | -0.177 |
| P value | 0.246 | 0.230 | 0.349 |
| Data presented as Pearson correlation coefficient (r) and p value. hsCRP: High-sensitivity c-reactive protein. TGF: Transforming growth factor. G-CSF: Granulocyte colocy stimulating factor. IFN: interferon. IL: interleukin. MIP: monocyte chemotactic protein. TNF: Tumor necrosis factor. | | | |

| Supplementary table 4. PP analysis of systemic cytokine concentrations and fibrosis markers before and after low-carbohydrate or low-fat diets and changes within and between groups. | | | | | | | | |
| --- | --- | --- | --- | --- | --- | --- | --- | --- |
|  | **BL** | **W9** | **Difference from BL to w9** | | | **Difference in change between groups** | | |
|  | **Mean ± SD** | **Mean ± SD** | **EMM** | **95% CI** | **P value** | **EMM** | **95% CI** | **P value** |
| hsCRP, mg/L |  |  |  |  |  |  |  |  |
| LCD | 3.1 ± 3.7 | 2.2 ± 2.1 | -1.3 | -2.3 to -0.3 | **0.010** | -0.2 | -1.7 to 1.2 | 0.743 |
| Low-fat diet | 5.6 ± 5.0 | 4.3 ± 5.2 | -1.1 | -2.2 to 0.0 | 0.061 |  |  |  |
| TGF-ß1, pg/ml |  |  |  |  |  |  |  |  |
| LCD | 11292.8 ± 3181.8 | 11481.7 ± 4317.7 | -705.9 | -2297.2 to 885.4 | 0.385 | -27.78 | -2148.8 to 2093.2 | 0.98 |
| Low-fat diet | 13951.6 ± 4357.0 | 12396.1 ± 4357.1 | -678.1 | -2431.1 to 1074.9 | 0.448 |  |  |  |
| TGF-ß2, pg/ml |  |  |  |  |  |  |  |  |
| LCD | 1451.3 ± 315.4 | 1542.0 ± 250.6 | 36.2 | -70.6 to 143.0 | 0.506 | 34.6 | -109.1 to 178.2 | 0.637 |
| Low-fat diet | 1633.9 ± 275.5 | 1582.2 ± 276.7 | 1.7 | -116.1 to 119.4 | 0.978 |  |  |  |
| TGF-ß3, pg/ml |  |  |  |  |  |  |  |  |
| LCD | 181.0 ± 59.9 | 182.8 ± 72.4 | -13.0 | -40.0 to 14.1 | 0.347 | -13.7 | -50.2 to 22.8 | 0.462 |
| Low-fat diet | 225.4 ± 70.9 | 213.0 ± 84.0 | 0.7 | -29.1 to 30.5 | 0.962 |  |  |  |
| Eotaxin, pg/ml |  |  |  |  |  |  |  |  |
| LCD | 32.0 ± 11.3 | 28.7 ± 8.3 | -3.0 | -5.2 to -0.7 | 0.012 | -1.9 | -5.2 to 1.5 | 0.277 |
| Low-fat diet | 27.6 ± 9.6 | 27.8 ± 9.2 | -1.1 | -3.7 to 1.5 | 0.412 |  |  |  |
| G-CSF, pg/ml |  |  |  |  |  |  |  |  |
| LCD | 15.5 ± 10.1 | 15.1 ± 11.4 | -0.5 | -3.7 to 2.7 | 0.749 | -4.7 | -9.5 to 0.1 | 0.053 |
| Low-fat diet | 26.4 ± 27.2 | 28.7 ± 24.1 | 4.2 | 0.6 to 7.8 | 0.023 |  |  |  |
| IFN-g, pg/ml |  |  |  |  |  |  |  |  |
| LCD | 3.3 ± 3.1 | 4.6 ± 3.5 | 1.0 | -0.9 to 2.9 | 0.305 | 0.5 | -2.4 to 3.3 | 0.749 |
| Low-fat diet | 11.4 ± 25.0 | 11.9 ± 21.3 | 0.5 | -1.6 to 2.6 | 0.628 |  |  |  |
| IL-4, pg/ml |  |  |  |  |  |  |  |  |
| LCD | 0.6 ± 0.4 | 0.6 ± 0.3 | -0.1 | -0.2 to 0.1 | 0.428 | -0.1 | -0.3 to 0.2 | 0.593 |
| Low-fat diet | 0.9 ± 1.0 | 0.9 ± 0.9 | -0.0 | -0.2 to 0.2 | 0.995 |  |  |  |
| IL-8, pg/ml |  |  |  |  |  |  |  |  |
| LCD | 1.1 ± 0.6 | 1.0 ± 0.7 | -0.1 | -0.4 to 0.1 | 0.364 | -0.2 | -0.6 to 0.2 | 0.261 |
| Low-fat diet | 1.9 ± 3.0 | 1.9 ± 2.5 | 0.1 | -0.2 to 0.4 | 0.495 |  |  |  |
| IL-9, pg/ml |  |  |  |  |  |  |  |  |
| LCD | 293.0 ± 46.2 | 281.5 ± 55.8 | -16.9 | -34.1 to 0.2 | 0.053 | -13.7 | -39.0 to 11.5 | 0.287 |
| Low-fat diet | 309.2 ± 110.0 | 306.6 ± 103.5 | -3.2 | -22.6 to 16.1 | 0.744 |  |  |  |
| IL-13, pg/ml |  |  |  |  |  |  |  |  |
| LCD | 5.9 ± 11.0 | 5.0 ± 11.7 | -0.6 | -1.5 to 0.3 | 0.213 | -0.5 | -1.8 to 0.9 | 0.491 |
| Low-fat diet | 5.4 ± 3.9 | 5.1 ± 3.3 | -0.1 | -1.1 to 0.9 | 0.850 |  |  |  |
| IP-10, pg/ml |  |  |  |  |  |  |  |  |
| LCD | 250.8 ± 100.9 | 230.8 ± 89.7 | -24.8 | -51.1 to 1.5 | 0.065 | -25.9 | -64.6 to 12.7 | 0.189 |
| Low-fat diet | 297.7 ± 163.4 | 297.5 ± 119.7 | 1.1 | -28.6 to 30.8 | 0.940 |  |  |  |
| MCP-1(MCAF), pg/ml |  |  |  |  |  |  |  |  |
| LCD | 9.5 ± 3.9 | 8.8 ± 3.4 | -1.0 | -2.1 to 0.0 | 0.059 | -1.6 | -3.2 to -0.1 | 0.040 |
| Low-fat diet | 10.2 ± 6.3 | 10.8 ± 6.4 | 0.6 | -0.6 to 1.8 | 0.313 |  |  |  |
| MIP-1α, pg/ml |  |  |  |  |  |  |  |  |
| LCD | 0.6 ± 0.3 | 0.5 ± 0.3 | -0.0 | -0.1 to 0.0 | 0.397 | -0.1 | -0.3 to -0.0 | 0.024 |
| Low-fat diet | 0.8 ± 0.6 | 0.9 ± 0.5 | 0.1 | 0.0 to 0.2 | 0.028 |  |  |  |
| MIP-1ß, pg/ml |  |  |  |  |  |  |  |  |
| LCD | 159.0 ± 14.3 | 151.7 ± 20.0 | -7.9 | -13.4 to -2.5 | **0.004** | -7.0 | -14.7 to 0.7 | 0.074 |
| Low-fat diet | 158.7 ± 14.9 | 158.0 ± 20.3 | -0.9 | -7.0 to 5.2 | 0.772 |  |  |  |
| RANTES, pg/ml |  |  |  |  |  |  |  |  |
| LCD | 2237.0 ± 339.5 | 2100.2 ± 453.7 | -98.5 | -221.6 to 24.6 | 0.117 | -106.6 | -282.1 to 68.9 | 0.234 |
| Low-fat diet | 2059.5 ± 394.4 | 2102.3 ± 423.2 | 8.1 | -130.6 to 146.9 | 0.909 |  |  |  |
| TNF-α, pg/ml |  |  |  |  |  |  |  |  |
| LCD | 47.8 ± 11.6 | 43.4 ± 9.1 | -5.4 | -9.0 to -1.9 | **0.003** | -4.9 | -10.1 to 0.3 | 0.063 |
| Low-fat diet | 54.5 ± 18.4 | 53.0 ± 20.3 | -0.5 | -4.5 to 3.5 | 0.791 |  |  |  |
| Data presented as mean ± SD, and results from linear mixed models are presented as estimated marginal means (EMM), 95% CI and p value. n = 35 in LCD and n = 35 in low-fat diet group at baseline, and n = 30 in LCD and n = 25 in low-fat diet group at w9. LCD: low-fat diet. hsCRP: High-sensitivity c-reactive protein. TGF: Transforming growth factor. G-CSF: Granulocyte colocy stimulating factor. IFN: interferon. IL: interleukin. MIP: monocyte chemotactic protein. TNF: Tumor necrosis factor. | | | | | | | | |


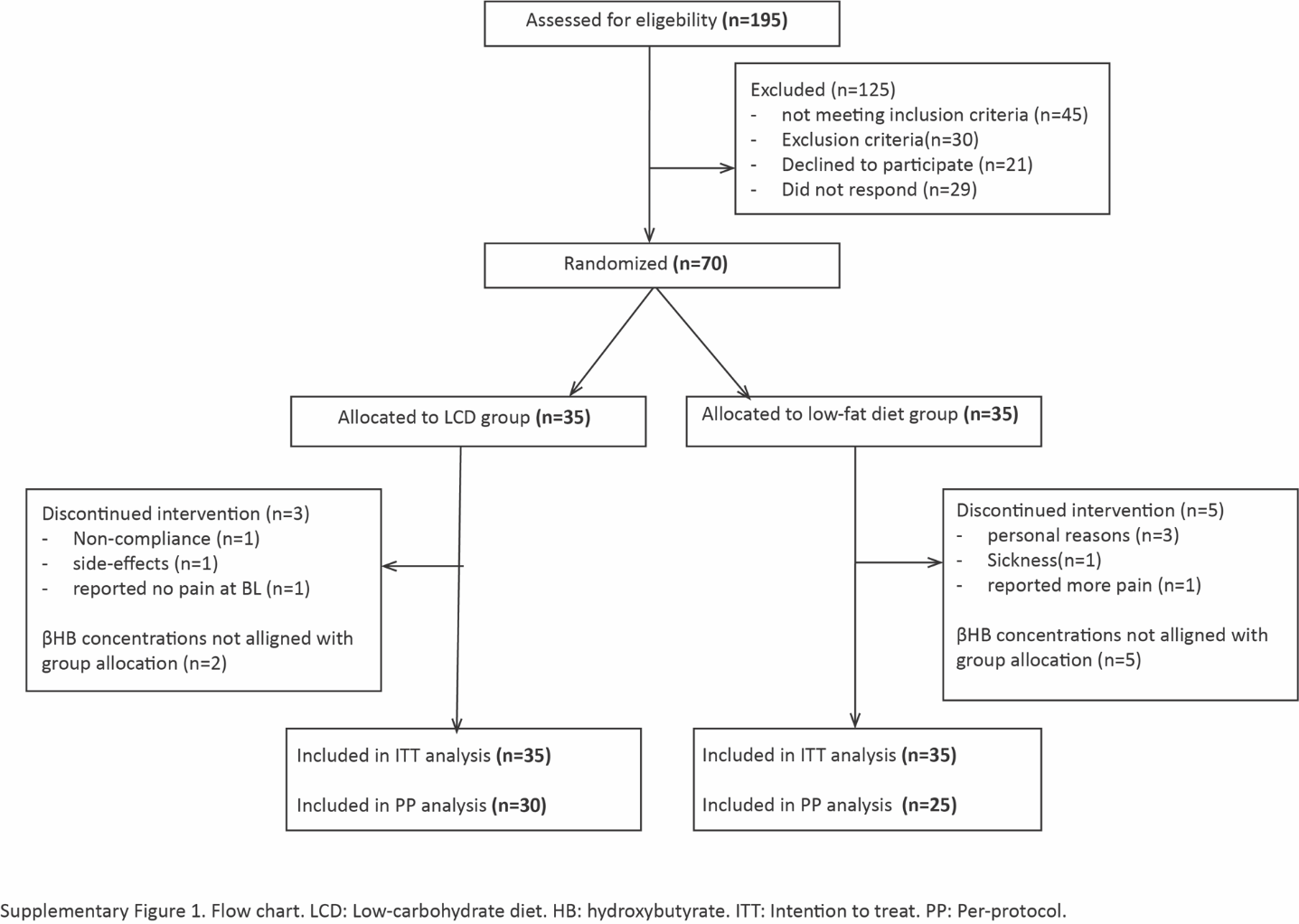

Supplement: Multimedia component 1 [file mmc1.docx]
